# A Good Gold Foil

**Published:** 1881-12

**Authors:** 


*A Good Gold Foil.*—We have learned to be cautious of new things and new manufacturers. Time was when we bought most everything that came out, but found that it was not always the newest that was best. Mr. Ed. Rowan, of Jersey City, left with the writer some time ago a book of gold foil of which we confess to have felt so suspicious as to lay it aside unused for a while. But on trial it was really an excellent make of gold, so much so that an ounce was bought, which proved equally good. We give this notice entirely unsolicited by Mr. R., who may be surprised to find his name in print, but we feel that he deserves it for making a really good gold foil. H.

